# Inter- and Intra-Observer Concordance of Cyberpathology in Twenty-Five Cases

**Published:** 2008-03

**Authors:** Tommy R. Tong, Kam-cheong Lee, Olivia Wai-hing Chan, Ka-leung Au, Wilson Man-shan Tsui, Genevieve M. Learmonth, Kelvin Ying-wai Leung, Cecilia Siu-nga Wong, Jessica Pik-man Lam

**Affiliations:** 1 *Department of Pathology, Princess Margaret Hospital, Hong Kong, China;*; 2 *Department of Pathology, Alice Ho Miu-ling Nethersole Hospital, Hong Kong, China;*; 3 *Department of Pathology, Caritas Medical Centre, Hong Kong, China;*; 4 *Department of Pathology, Groote Schuur Hospital, and University of Cape Town, South Africa*

**Keywords:** cyberpathology, telecytology, telepathology, internet

## Abstract

To study the feasibility of anatomical pathology consultation in cyberspace (cyberpathology) and to determine inter- and intra-observer concordance. Twenty-five consecutive cytology and histopathology cases are photographed using a digital camera placed against the eyepiece, and uploaded to an image-server in the Internet. Participants view the images, rate their confidence, and provide a diagnosis. They then view the original glass slides and provide a final diagnosis. The diagnoses are compared for inter- and intra-observer concordance. Participants are confident of their diagnoses based on viewing images on the Internet. The intra-observer concordance exceeds 95% individually, and 96% overall. Inter-observer concordance was 100% in a subset of cases. Cyberpathology as described is both available and affordable and is a valid alternative to slide-based anatomic pathology consultation.

## INTRODUCTION

The practice of cytopathology and histopathology at a distance require the sharing of glass slides. A bottleneck exists at the level of slide transfer. In addition, cytology slides are often irreplaceable and cannot be sent to more than one recipient at a time. With the increasing inter-connectedness of the world and the arrival of multimedia-enabled Internet technology, this bottleneck is being assaulted from all sides. The impact of information technology on histopathology is recently reviewed (1-3)

Telecytology and telepathology utilizing various direct network connections as well as the Internet has been the subject of many studies (4-6). However, to date it is not widely practiced except in experimental and pioneer settings. The reasons include the reluctance to view images on the monitor, the complexity and cost of initial setup and maintenance of the hardware and software, the difficulty of digitizing images, security issues and concerns over the accuracy of opinions rendered on digital images. The first is simply a habitual hurdle that endoscopists and laparoscopists have overcome - few continue to peer down the endoscope or laparoscope; they choose instead to adopt the more ergonomic way of viewing monitor screens hooked up to their scopes. Cytological and histopathological diagnosis entails examination of representative samples of a given specimen. This also applies to viewing images of tissue sections on paper or monitor in much the same way as an electron microscopist would. Obviously, the number of images viewed has an impact on the confidence of the pathologist and this is another hurdle that needs to be overcome by technology if telepathology were to prosper. Another major hurdle is in finding a solution to the complexity and cost of the overall process. These are some of the problems the authors faced when trying to practice telepathology.

We describe a simple and economical telepathology setup that is easy to use, and takes advantage of freely available third-party Internet hosting service for images. We found the low cost, high quality, consumer type digital camera a very useful tool to cut down on the cost of the overall setup. In addition, we showed that pathologists were able to render accurate diagnoses based on viewing samples of digital images.

## MATERIALS AND METHODS

The hardware employed include a Nikon Labophot-2 microscope, an Olympus C2000Z digital camera, a Pentium II MMX IBM 300GL desktop computer with 192MB RAM and a LAN connection with Internet access behind a firewall. The software used includes Internet browser and imaging software that came with the digital camera (Camedia Master™ 1.1).

Twenty five consecutive cases of biopsies, surgical pathology specimens, bone marrow aspirate and fine needle aspiration cytology specimens that the author deemed to require a second opinion or an expert consultation in a normal pathology practice were collected over a 15-week period. Several pathologists participated, including two hematopathologists, who viewed the hematology cases for inter-observer concordance.

Selected areas of diagnostic value, including random fields of various magnifications, were captured digitally at standard or high resolution in JPEG format using the method of Tse *et al* (7). Briefly, the camera was set to automatic exposure, aperture and focus. The flash was disabled. With the area of interest in focus, the camera lens was applied against one of the eyepieces and the image as seen in the LCD display of the camera assessed by the naked eye for adequacy of focus and illumination. The 'peeping hole' effect was abolished using digital zoom to fill up the CCD or simply ignored in most cases. When the image appeared satisfactory, the shutter was pushed without shaking the camera.

After the required images were photographed, they were transferred to the physical memory (hard-disk) of the computer by using the serial cable of the camera or by removing the storage medium (Smartmedia™ card) in the camera and downloading it to the computer with the help of a low cost card reader (Filand™). The camera named images serially at the time of photography. In cases where the images need to be designated, the file names were changed manually. The quality of the digital images was determined empirically. Only images judged to be of sufficient quality or relevance are included in the study.

With an active Internet connection, an account was first setup at http://www.kodakgallery.com by visiting the website and registering for the free service. The information required for registration included a valid email address, a user-defined password and the user’s firstname.

After completing the registration, the images were uploaded using one of two user-friendly methods (browser upload or email attachment). The server processed the files and presented them at the “new” album. The images were then annotated or manipulated by following simple instructions. Image quality was again determined empirically and found to be satisfactory.

Participants were then notified by clicking the “Share photos” tab followed by entering the participants’ email addresses and a message. The participants received emails with a hyperlink to the album. By clicking on the hyperlink, the album was displayed on the window of the Internet browser at the participants’ computer monitors. Clicking the thumbnail resulted in the display of the full sized image. A nice feature was “slideshow”, which reduces significantly the number of mouse clicks, and hence user fatigue, if there were many images. The time taken to download the images was determined by the speed of the Internet connection.

**Figure 1:**
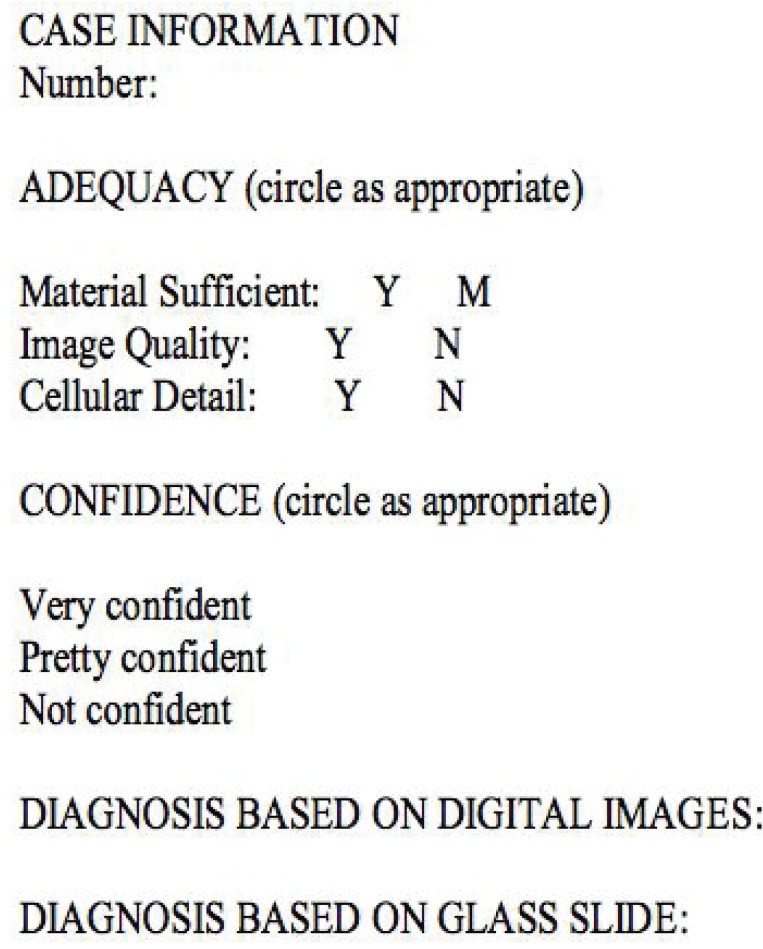
The proforma used in this study, as adapted from reference 8.

**Figure 2:**
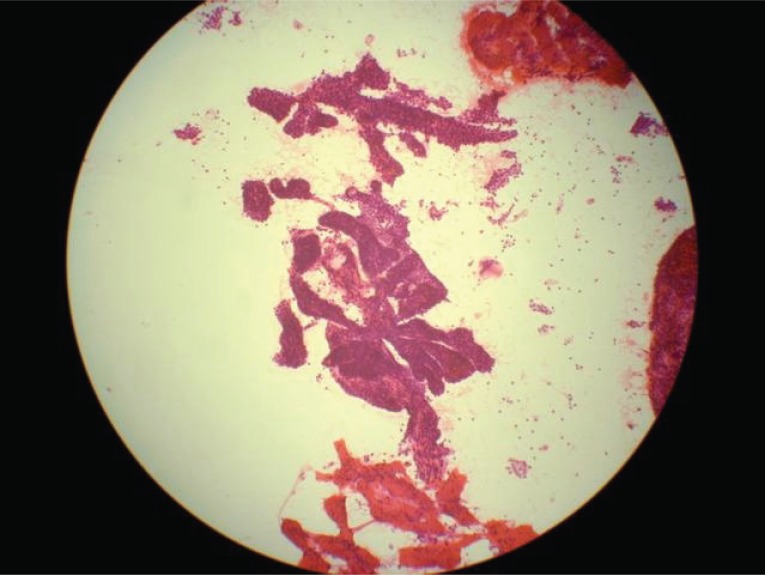
Low magnification of H&E stained fine needle aspiration of papillary carcinoma of thyroid (case 2), depicting papillary fronds of neoplastic cells.

Participants were given a proforma to complete (Fig. 1). The proforma was adapted from Figure 2 of a previous work (8). The participants filled out designated fields after viewing the images on the Internet. They were then given the glass slides. After viewing the slides, the final diagnosis was written in a separate box.

The inter- and intra-observer concordance of diagnoses based on images and glass slides were analyzed. Also analyzed were responses to 'adequacy' and 'confidence'.

## RESULTS

**Figure 3:**
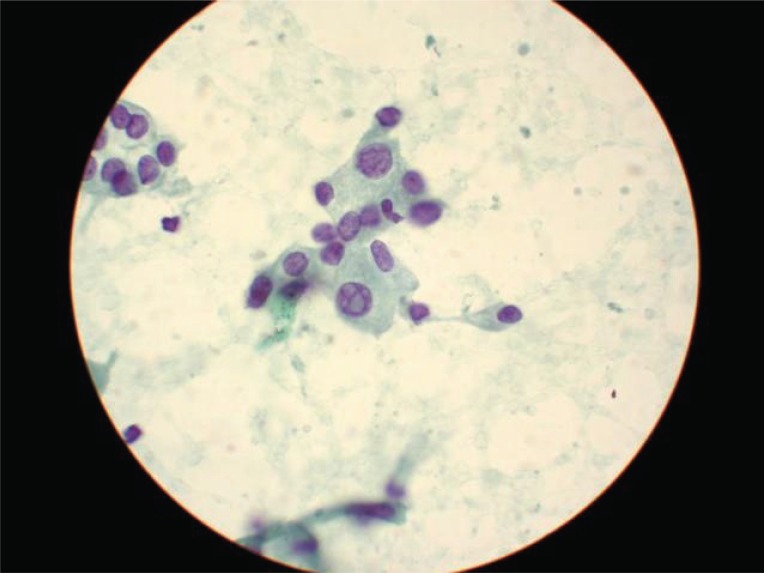
High magnification of PAP stained fine needle aspiration of papillary carcinoma of thyroid (case 2), illustrating intranuclear pseudoinclusion (center), among other nuclear features of papillary carcinoma.

An average of 18 images were presented per case. The images of all 25 cases, except case 14 were judged satisfactory for evaluation (Table 1). The images from case 14 were of suboptimal quality and led to a wrong initial diagnosis of cytomegalovirus (CMV) infection in a case with adenocarcinoma. However, software enhancement (by adjusting contrast and brightness) of the digital images without re-photographing the case followed by re-evaluation by the same participant anonymously 3 months later resulted in a correct diagnosis. The concordance between diagnoses rendered on viewing the cyberimages and diagnoses based on viewing glass slides were 96 % and 100 %, before and after image enhancement (case 14). Otherwise, there was no major discrepancy between the image-based diagnoses and the slide-based diagnoses (Table 2). Figures 2 and 3 are examples of digital images as viewed by participants. Images used for this study can be accessed at the following website: www.kodakgallery.com. Login using dr10029@hotmail.com; password: 10029. After logging in enter the following URL in your browser and click a case number: www.geocities.com/tommytongmd/telepath/telepath.htm.

**Table 1:** Adequacy of images

Nature of specimen	Material sufficient	Image quality	Cellular detail
	Yes/No/no answer	Yes/No/no answer	Yes/No/no answer

Biopsy (n=13)	11/0/2	11/0/2	10/0/3
FNA cytology (n=5)	5/0/0	5/0/0	4/1/0
Surgical excision (n=6)	5/0/1	5/0/1	5/0/1
Bone marrow smear (n=1)	1/0/0	1/0/0	1/0/0

**Table 2:** Intra-observer concordance and level of confidence

Case no.(B/C/S/M)^a^	Diagnosis - image	Diagnosis - slide	Confidence (V, P or N)^b^	Remarks

1 (S)	Benign. Fibrous dysplasia is in the differential.	Fibrous dysplasia	P	
2 (C)	Papillary thyroid carcinoma	Papillary thyroid carcinoma	V	
3 (B)	Adenocarcinoma	Adenocarcinoma	P	
4 (B)	Primary biliary cirrhosis	Primary biliary cirrhosis	P	
5 (C)	Suspicious for adenocarcinoma	Adenocarcinoma	P	
6 (M)	Megaloblastic anemia	Megaloblastic anemia	V	
7 (B)	Desmoid fibromatosis	Desmoid fibromatosis	P	
8 (B)	Adenocarcinoma, poorly differentiated, with focal squamous area	Same	P	
9 (B)	Cirrhosis	Cirrhosis, hepatitis C-related	V	
10 (B)	Erythema nodosum	Erythema nodosum	P	
11 (C)	Hodgkin’s disease, lymphocyte predominant	Hodgkin’s disease, lymphocyte predominant	P	Subsequent excision - Hodgkin’s disease, nodular sclerosis.
12 (S)	Synovial sarcoma	Synovial sarcoma	V	
13 (B)	Metastatic undifferentiated carcinoma	Metastatic undifferentiated carcinoma	P	Later nasopharyngeal biopsy-lymphoepithelioma-like carcinoma
14 (B)	CMV, ulcer	Adenocarcinoma	P	Poor image quality
14 (B)	Adenocarcinoma (second attempt 3 months later)	Adenocarcinoma	P	After software enhancement of images
15 (B)	Multiple myeloma	Multiple myeloma	V	
16 (B)	Cirrhosis,active	Cirrhosis, active	P	
17 (S)	Dermatofibroma	Dermatofibroma	P	
18 (B)	Chronic hepatitis with fibrosis, ? cirrhosis, ? autoimmune	Autoimmune hepatitis	P	
19 (S)	Fungal infection, ? protothecosis.	Protothecosis	V	
20 (S)	Hodgkin’s disease	Hodgkin’s disease, nodular sclerosis	V	
21 (C)	Squamous cell carcinoma	Squamous cell carcinoma	P	
22 (C)	Pleomorphic adenoma	Pleomorphic adenoma	P	
23 (B)	Endometrioid adenocarcinoma	Endometrioid adenocarcinoma	P	
24 (B)	Carcinoid tumor	Carcinoid tumor	P	
25 (S)	Pulmonary blastoma	Pulmonary blastoma. Carcinosarcoma or pulmonary blastoma	P	

a B/C/S/M, Biopsy/FNA cytology/Surgical Excision/Bone marrow smear;

b V/P/N, Very confident/Pretty confident/Not confident.

The intra-observer concordance ranges from 95% (21 of 22 cases) to 100%. Inter-observer concordance was evaluated for the hematology cases, with 100% agreement between three participants.

Individuals differed in their use of “very confident” and “pretty confident”. None of the participants were “not confident” about their image-based diagnoses. Most participants remarked that they adjusted quickly to rendering image-based diagnosis.

The same degree of accuracy was seen in the fine needle aspiration cytology cases as the surgical or biopsy cases. When additional studies such as immunohistochemistry were performed (cases 12, 15, 17, 20, 24 and 25), participants commented that they were of value in arriving at an accurate classification or diagnosis of a clonal disease (multiple myeloma, case 15).

## DISCUSSION

Pathologists are keen on exploiting digital technology. Telepathology comes in two main forms. The most common form, as employed in the current study, consists of a static, non-interactive presentation of information and images (9-11). The other form of telepathology is dynamic, real-time, and interactive (5, 12-16). In robotic telepathology, motorized microscopes operated by remote computer users in real-time are employed. However, there are hardware limitations as well as time-zone differences to address for this methodology, not to mention the costs involved. If there is more than one slide, a person needs to stand by the microscope to change slides. This method is ill suited for locations with significant time zone differences. Alternative to robotic telepathology, real-time telepathology are also practiced using web-based video-conferencing technology (17, 18). Virtual microscopy, employing fully digitized glass slides, serves to replace the microscope but has its own limitations in the setting of pathology consultation.

The technique used in the current study is made possible by the use of digital photography using consumer grade digital camera and proprietary “dot.com” innovations in sharing images on the Internet. The advantage is that it combines the ease of use of the digital camera, and the ability to share images with any number of invited consultants. This ability to share images is particularly suited for external quality assurance activity in that all participants see exactly the same images.

This study started with skeptical participants whose confidence increased with use. Although the images were of low resolution, a target diagnosis could still be made for both histological and cytological materials. Both common and rare cases were diagnosed accurately based on viewing representative images on the computer monitor (Table 2). Our experience agrees with published results of similar studies (19, 20).

The number of images appeared to be important in histopathological as well as in cytological materials. One of the 5 FNA cytology cases (case 5) was diagnosed as “suspicious for adenocarcinoma” based on the 13 images. The suspicion might be removed if more images had been submitted.

Case 11 (Hodgkin lymphoma) showed minor discrepancy between the cytological and histopathological diagnosis. This phenomenon is observed in cytology owing to limitation of sampling rather than being a problem specific to telecytology. The concordance between the telecytology diagnoses and the glass slide diagnoses testifies to the argument. Three participants saw the hematology cases and case 25 and all arrived at essentially the same conclusions based on viewing the images. These cases addressed inter-observer concordance, which was also studied by others (21-23). When inter-observer and intra-observer concordances are compared, the former shows larger discrepancy (16).

Because any number of participants may be invited to view exactly the same images, this setup is suitable for quality assurance activities such as mandatory second opinion (24). Virtual microscopy of digital slides on other media such as optical disk may also be suitable for this kind of activity (19).

For Internet security, patients in this study were identified by case numbers. If patient demographics need to be transmitted to the remote user, security can be assured by using facsimile or separate email employing encryption technology (20).

This study demonstrates that histopathological or cytological diagnosis is possible based on viewing representative static images on a computer monitor. The high intra-observer concordance (100%) compares favorably with that of other studies, which range from 80% to 100% (12, 18, 24-32). This study further described an easy and economical cyberpathology setup that demands minimal knowledge of the workings of the Internet and a small investment in hardware. In addition to consultation, we think this method is also suitable for external quality assurance activities.
